# Plasma alpha-trypsin inhibitor heavy chain 4 as an age-specific biomarker in the diagnosis and treatment of major depressive disorder

**DOI:** 10.3389/fpsyt.2024.1449202

**Published:** 2024-09-11

**Authors:** Ping He, Xuefang Lu, Mengmeng Zhong, Hui Weng, Jialu Wang, Xiaoxuan Zhang, Chen Jiang, Feng Geng, Yachen Shi, Gaojia Zhang

**Affiliations:** ^1^ Department of Neurosurgery Intensive Care Unit, The Affiliated Wuxi People’s Hospital of Nanjing Medical University, Wuxi People’s Hospital, Wuxi Medical Center, Nanjing Medical University, Wuxi, China; ^2^ Department of Neurology, The Affiliated Wuxi People’s Hospital of Nanjing Medical University, Wuxi People’s Hospital, Wuxi Medical Center, Nanjing Medical University, Wuxi, China; ^3^ Department of Rehabilitation Medicine, The Affiliated Wuxi People’s Hospital of Nanjing Medical University, Wuxi People’s Hospital, Wuxi Medical Center, Nanjing Medical University, Wuxi, China; ^4^ Department of Functional Neurology, The Affiliated Wuxi People’s Hospital of Nanjing Medical University, Wuxi People’s Hospital, Wuxi Medical Center, Nanjing Medical University, Wuxi, China; ^5^ Department of Psychology and Sleep Medicine, The Second Hospital of Anhui Medical University, Hefei, China; ^6^ Department of Interventional Neurology, The Affiliated Wuxi People’s Hospital of Nanjing Medical University, Wuxi People’s Hospital, Wuxi Medical Center, Nanjing Medical University, Wuxi, China; ^7^ Department of Neurosurgery, The Affiliated Wuxi People’s Hospital of Nanjing Medical University, Wuxi People’s Hospital, Wuxi Medical Center, Nanjing Medical University, Wuxi, China

**Keywords:** major depressive disorder, inter-alpha-trypsin inhibitor heavy chain 4, glial fibrillary acidic protein, astrocyte, antidepressive treatment

## Abstract

**Background:**

The diagnosis of major depressive disorder (MDD) mainly depends on subjective clinical symptoms, without an acceptable objective biomarker for the clinical application of MDD. Inter-alpha-trypsin inhibitor heavy chain 4 (ITIH4) showed a high specificity as biomarker for the diagnosis and treatment of MDD. The present study aimed to investigate differences in plasma ITIH4 in two different aged MDD patients and underlying pathological mechanisms of plasma ITIH4 in the occurrence and development of MDD.

**Methods:**

Sixty-five adult MDD patients, 51 adolescent MDD patients, and 64 healthy controls (HCs) were included in the present study. A 14-days’ antidepressive treatment was conducted in all MDD patients. Psychological assessments were performed and plasma ITIH4 and astrocyte-related markers were detected for all participants.

**Results:**

(1) Plasma levels of ITIH4 in adult MDD patients were significantly higher than adolescent MDD patients and HCs, and significantly increased plasma ITIH4 levels was observed in adolescent MDD patients compared with HCs (2). There were positive correlations between plasma ITIH4 levels and 24-item Hamilton Depression Scale (HAMD-24) scores and plasma glial fibrillary acidic protein (GFAP) levels in MDD patients, however, plasma ITIH4 levels were significantly correlated with age just in adult MDD patients (3). Plasma ITIH4 showed area under the curve values of 0.824 and 0.729 to differentiate adult MDD patients and adolescent MDD patients from HCs, respectively (4). There was significant decrease in plasma levels of ITIH4 between before and after antidepressive treatment in adult MDD patients, but not in adolescent MDD patients (5). Changed value of ITIH4 levels were correlated with the changed value of GFAP levels and changed rate of HAMD-24 scores in adult MDD patients following antidepressive treatment.

**Conclusion:**

Plasma ITIH4 may be potential plasma biomarkers of MDD with age-related specificity, which was associated with depressive symptoms astrocyte-related pathologic changes, and antidepressive treatment efficacy.

## Introduction

Major depressive disorder (MDD) is the most common mental disorder, with an increasing yearly prevalence (1, 2). The primary clinical symptoms of MDD include a depressed mood, anhedonia, and suicidal thoughts (3). Although the precise pathological mechanism of MDD is still unclear, several hypotheses are proposed to explain the underlying basis of MDD, including the monoamine hypothesis, the hypothalamic-pituitary-adrenal axis dysfunction, neuroinflammatory, the genetic and epigenetic anomaly (4). Currently, the diagnosis of MDD mainly depends on subjective recognition of the clinical symptoms, without objective diagnostic evidence (5–7). Therefore, identification of effective biomarker for the clinical application of MDD is essential. Blood-based indicators could be used as convenient tools to improve the accuracy of clinical diagnosis in MDD, such as brain-derived neurotrophic factor (8, 9). However, the widely accepted objective biomarker for the diagnosis of MDD is still lacking. Meanwhile, dissatisfactory accuracy of identification is also the common issue for researches of biomarkers in MDD, which may be affected by the individual characteristics, *e.g.*, sex (10, 11). Exploration of potential influencing factors of biomarkers is essential for the utilization of them in individualized diagnosis and treatment of MDD.

Inter-alpha-trypsin inhibitor heavy chain 4 (ITIH4) is a 120 kDa plasma glycoprotein, belonging to the liver-restricted serine protease inhibitor family (12). ITIH4, as an acute phase protein, can play a key role of anti-inflammation in neuroinflammation (13, 14). Two studies of proteomic analysis found that ITIH4 levels was significantly elevated in the serum protein profiling of MDD patients when compared to health controls (15, 16). Our previous study firstly revealed the plasma ITIH4 was a diagnostic biomarker of MDD with high specificity and consistent expression of ITIH4 were verified between plasma and the post-mortem brain tissue in MDD patients (17). Another study based on post-mortem brain tissue found that ITIH4 gene expression was up-regulated in the prefrontal cortices of MDD patients using DNA microarray (18). Moreover, genetic variants of the ITIH4 genes were identified to be associated with the higher risk of suicidal attempts in mood disorders (19). These evidences support that ITIH4 may be involved in the pathology of MDD and peripheral ITIH4 may be used as a valuable diagnostic indicator of MDD. Furthermore, in another of our studies, plasma ITIH4 levels were significantly reduced at the end of repetitive transcranial magnetic stimulation treatment as compared to before treatment (20). Hence, plasma ITIH4 may be also a potential index for evaluating the effect of antidepressive treatment. However, all previous studies were performed in adult MDD patients, and whether individual characteristics, *e.g.*, sex, age, can affect the levels of plasma ITIH4 is unclear.

In the present study, we aimed to assess differences in plasma ITIH4 among HCs, adult MDD patients, and adolescent MDD patients. Potential associations between plasma ITIH4 levels and clinical characteristics of MDD patients were also explored. Additionally, the clinical value of plasma ITIH4 in the treatment of antidepressive drugs in MDD patients was thoroughly assessed.

## Materials and methods

### Study cohorts

There were two cohorts were included in the present study. Cohort one included 65 adult MDD patients and 64 healthy controls (HCs), and was recruited from the Affiliated Wuxi People’s Hospital of Nanjing Medical University. Cohort two consisted of 51 adolescent MDD patients recruited from the Second Affiliated Hospital at Anhui Medical University.

All participants or their legal guardians provided informed consent. The ethical approval was obtained from the Ethics Committee of the Affiliated Wuxi People’s Hospital of Nanjing Medical University (approval number: KY22082). The study was also approved by the Ethics Committees of the Second Hospital of Anhui Medical University (approval number: SL-YX2024-022).

### Inclusion and exclusion criteria of participants

The inclusion criteria for MDD patients included the following: (1) first episode outpatients or inpatients; (2) patients that were drug-naïve or drug-free (*i.e.*, free of antidepressive treatment longer than 4 weeks prior to the beginning of the study); (3) no family history of psychosis. Meanwhile, adult MDD patients were aged 18 to 55 years, and adolescent MDD patients were aged 12 to 17 years. Furthermore, HCs had no history of DSM-V Axis I disorders, mental disorders or severe physical disease.

Participants fulfilling any of the following criteria were excluded: (1) organic lesions of the central nervous system or neurodegenerative disorders, including stroke or Alzheimer’s disease; (2) secondary mental disorders, *e.g.*, those caused by the severe physical illness; (3) other types of psychiatric disorders other than MDD, *e.g.*, schizophrenia; (4) abuse and dependence of alcohol or drugs; (5) significant physical disorder (*e.g.*, endocrine disease, autoimmune disease, or impaired function of the liver or kidneys); or (6) any type of tumor or cerebral trauma.

### Treatment

All MDD patients, including adult and adolescent ones, obtained a 14-days’ antidepressive treatment. Only one kind of the selective serotonin reuptake inhibitors or serotonin-norepinephrine reuptake inhibitors was used as the primary antidepressant throughout the treatment. Furthermore, one kind of the atypical antipsychotics was additionally used when single antidepressant provided a poor efficiency, *e.g.*, aripiprazole, quetiapine. Detailed information of drugs was displayed in **Supplementary Material**. Notably, because assessment of antidepressive treatment efficacy was not the major goal of this study, the current study was designed as an observational study rather than a randomized control trail.

### Psychological assessments

The interviews of participants were completed by a trained researcher according to the Diagnostic and Statistical Manual of Mental Disorders, 5th Edition (DSM-V) (21). Meanwhile, the psychological assessments, including the 24-item Hamilton Depression Scale (HAMD-24) (22) and Self-Rating Depression Scale (SDS) (23), were also conducted to evaluate the depressive symptoms. The psychological assessments were performed for all MDD patients before and after treatment.

### Plasma sampling

After overnight fasting, peripheral venous blood was collected into EDTA-coated tubes. Within 30 minutes of collection, the blood samples were centrifuged at 1000 × g at 4°C for 10 minutes, subsequently plasma was aspirated and stored at –80°C until required for analysis. All MDD patients provided plasma samples before treatment, however, after the 14-days’ treatment, just 30 MDD patients provided plasma samples in adult MDD group and adolescent MDD group, respectively.

### Enzyme-linked immunosorbent assay analyses

The concentrations of plasma ITIH4, glial fibrillary acidic protein (GFAP), and S100beta protein (S100β) were measured in triplicate, using the commercial ELISA kits (FineTest, Wuhan, China; Catalog Number: EH1525 for ITIH4, EH0410 for GFAP, and EH0543 for S100β) in accordance with the manufacturer’s protocols. The concentration of protein in each plate was calculated according to standard curves and dilution factors. The inter- and intra-assay coefficients of variation were < 5%.

### Statistical analysis

Data were analyzed using SPSS version 22.0 (SPSS, Inc., Chicago, IL, USA). The Kolmogorov-Smirnov test was used to evaluate the normal distribution of the data. Categorical variables were analyzed using a chi-squared test. Continuous variables using a one-way ANOVA (Bonferroni correction for *post-hoc* test) when normally distributed, otherwise using a Kruskal-Wallis H test (Nemenyi correction for *post-hoc* test). The paired t-test was used for the comparison of changes of variables before and after intervention. Correlation analysis was performed to determine the relationships between two variables. Line regression analysis was used for the multivariate analysis. Receiver operating characteristic (ROC) curves were used to calculate the area under the curve (AUC) for determining the diagnostic accuracy of the plasma indices. The Youden index (24) was used to assess optimal values of sensitivity and specificity. Significance was defined as p < 0.05 (two tailed).

## Results

### Characteristics of participants

The demographic and clinical characteristics of participants across the three groups are summarized in **Table 1**. No significant differences were observed among these three groups in terms of sex and body mass index. In addition, there were there was no significant difference in duration of the disease between adult MDD and adolescent MDD groups. However, there was significant difference in age between adolescent MDD patients and HCs or adult MDD patients.

**Table 1:** Clinical characteristics and plasma indices levels of participants.

	HC (N = 64)	Adult MDD (N = 65)	Adolescent MDD (N = 51)	P_1_-value	P_2_-value	P_3_-value
Age, year	34.33 ± 10.58	33.49 ± 12.43	15.49 ± 1.22	1.000^#^	<0.001^#^	<0.001^#^
Sex, male%	26 (40.63%)	24 (36.92%)	13 (25.49%)	0.666^†^	0.089^†^	0.190^†^
BMI	23.29 ± 3.16	23.27 ± 3.54	22.06 ± 4.53	1.000^*^	0.244^*^	0.249^*^
Duration of the disease, month	–	32.92 ± 33.52	22.69 ± 24.13	–	–	0.224^$^
HAMD-24 scores	1.39 ± 1.74	19.66 ± 7.48	19.75 ± 6.97	<0.001^#^	<0.001^#^	1.000^#^
SDS scores	41.05 ± 8.81	69.42 ± 13.40	73.29 ± 9.74	<0.001^#^	<0.001^#^	0.657^#^
Plasma ITIH4 levels, ng/ml	1000.52 ± 195.77	1914.72 ± 780.92	1477.00 ± 722.75	<0.001^#^	<0.001^#^	0.040^#^
Plasma GFAP levels, ng/ml	0.17 ± 0.04	0.22 ± 0.11	0.22 ± 0.09	0.014^#^	0.021^#^	1.000^#^
Plasma S100β levels, pg/ml	77.01 ± 27.30	95.61 ± 22.86	93.34 ± 26.25	< 0.001^*^	0.002^*^	1.000^*^
SSRIs or SNRIs, N%						
Escitalopram	–	25 (38.46%)	14 (27.45%)	–	–	–
Sertraline	–	11 (16.92%)	23 (45.10%)	–	–	–
Duloxetine	–	15 (23.08%)	6 (11.76%)	–	–	–
Fluvoxamine	–	5 (7.69%)	6 (11.76%)	–	–	–
Fluoxetine	–	3 (4.62%)	2 (3.92%)	–	–	–
Paroxetine	–	6 (9.23%)	0 (0.00%)	–	–	–
Combined AAs, N%	–	27 (41.54%)	25 (49.02%)	–	–	–

Data are presented as means (standard deviation) or number of participants in each group (% of total).

HC, healthy control; MDD, major depressive disorder; BMI, body mass index; HAMD-24, 24-item Hamilton Depression Scale; SDS, Self-Rating Depression Scale; ITIH4, inter-alpha-trypsin inhibitor heavy chain 4; GFAP, glial fibrillary acidic protein; S100β, S100beta protein; SSRIs, selective serotonin reuptake inhibitors; SNRIs, serotonin-norepinephrine reuptake inhibitors; AAs, atypical antipsychotics.

P_1_-value: HC vs. Adult MDD; P_2_-value: HC vs. Adolescent MDD; P_3_-value: Adult MDD vs. Adolescent MDD.

^#^Kruskal-Wallis test (post hoc test: Nemenyi test); ^*^One-way ANOVA test (post hoc test: Bonferroni test); ^†^Chi-square test; ^$^Mann-Whitney U test.

Compared with HCs, adult MDD patients or adolescent MDD patients showed significantly increased HAMD-24 scores, SDS scores, plasma GFAP levels, and plasma S100β levels, however, no significant difference was found between adult MDD and adolescent MDD groups regarding these characteristics (**Table 1**). Furthermore, plasma ITIH4 levels were significantly different between each two of these groups (**Table 1**), and adult MDD patients exhibited the highest levels of plasma ITIH4, followed by adolescent MDD patients.

### Association of plasma ITIH4 with demographic and neuropsychological characteristics and plasma indices in MDD patients

As shown in **Table 2**, an elevated plasma ITIH4 level was correlated with higher age, HAMD-24 scores, plasma GFAP and S100β levels in adult MDD patients. The positive correlations between plasma ITIH4 and HAMD-24 scores and plasma levels of GFAP were also observed in adolescent MDD patients (**Table 2**). However, no significant correlation was observed between plasma levels of ITIH4 and age of the participants in adolescent MDD group.

**Table 2:** Correlation analyses of ITIH4 levels with clinical characteristics and plasma indices levels in adult MDD participants and adolescent MDD participants.

	Adult MDD	Adolescent MDD
Age	**R = 0.359; P = 0.003**	R = -0.088; P = 0.541
BMI	R = 0.081; P = 0.520	R = 0.082; P = 0.567
Duration of the disease	R = 0.082; P = 0.514	R = 0.042; P = 0.772
GFAP level	**R = 0.313; P = 0.011**	**R = 0.308; P = 0.028**
S100β level	**R = 0.247; P = 0.047**	R = 0.247; P = 0.080
HAMD-24 score	**R = 0.314; P = 0.011**	**R = 0.337; P = 0.016**
SDS score	R = 0.226; P = 0.070	R = 0.184; P = 0.195

HC, healthy control; MDD, major depressive disorder; BMI, body mass index; ITIH4, inter-alpha-trypsin inhibitor heavy chain 4; GFAP, glial fibrillary acidic protein; S100β, S100beta protein; HAMD-24, 24-item Hamilton Depression Scale; SDS, Self-Rating Depression Scale. The bold indicates significant results.

Further multivariate analysis indicated that plasma ITIH4, plasma GFAP, and age had significant and interactive effects on the HAMD-24 scores in adult MDD patients (**Table 3**). However, in the adolescent MDD group, just plasma ITIH4 could significantly affect the HAMD-24 scores using line regression analysis (**Table 3**).

**Table 3:** Line regression analysis for the association of HAMD-24 scores with the demographic characteristics and plasma indices levels in two MDD groups.

	Adult MDD	
	β	P-value
Age	-0.257	0.035
Sex	0.033	0.776
BMI	-0.136	0.253
ITIH4 level	0.308	0.016
GFAP level	0.460	<0.001
S100β level	-0.001	0.992
	Adolescent MDD	
	β	P-value
Age	-0.001	0.993
Sex	-0.052	0.738
BMI	-0.269	0.095
ITIH4 level	0.303	0.044
GFAP level	-0.117	0.447
S100β level	0.140	0.381

MDD, major depressive disorder; BMI, body mass index; HAMD-24, 24-item Hamilton Depression Scale; ITIH4, inter-alpha-trypsin inhibitor heavy chain 4; GFAP, glial fibrillary acidic protein; S100β, S100beta protein.

### Diagnostic performance of plasma ITIH4

**Figure 1:**
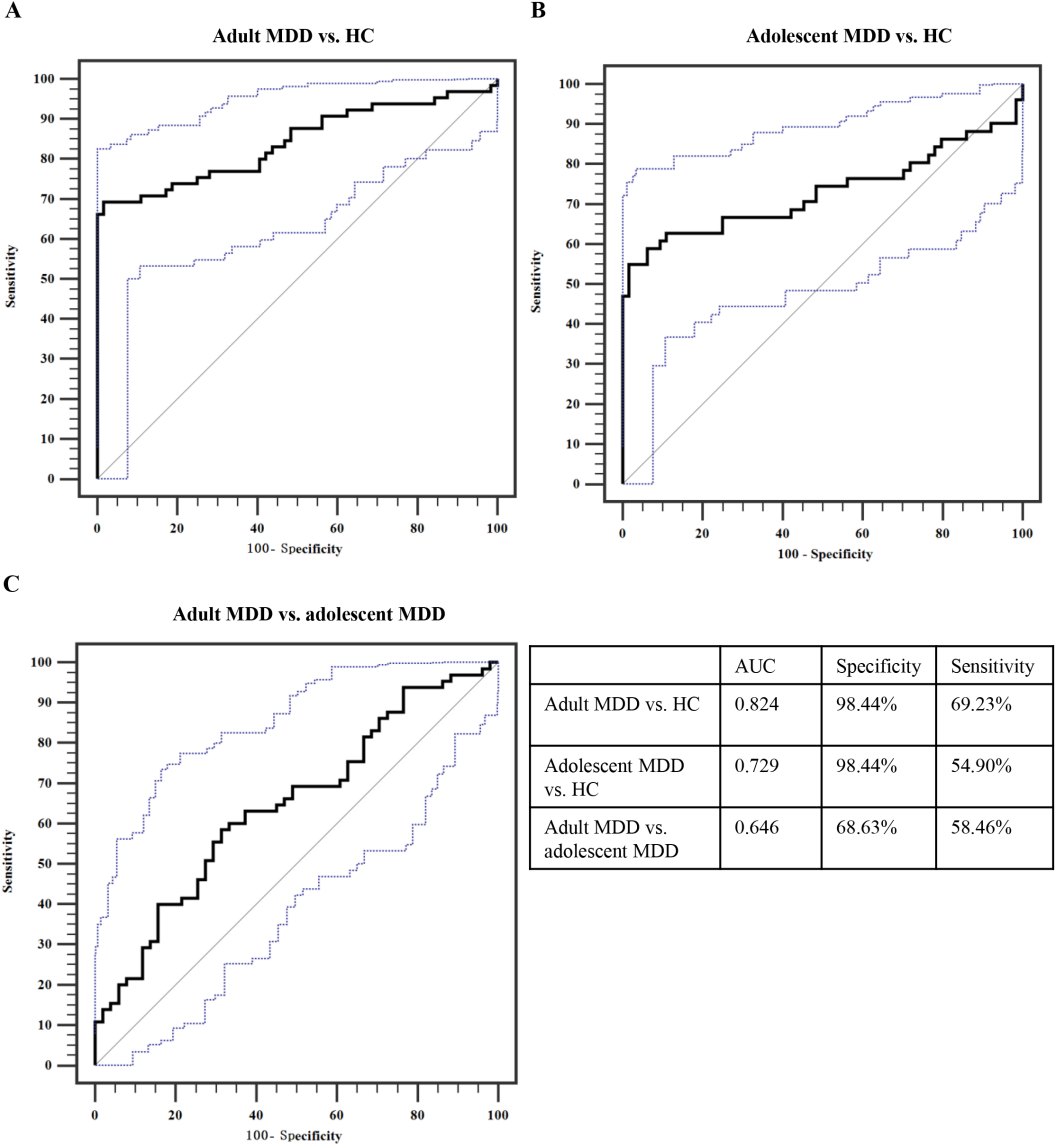
ROC curve analysis of plasma ITIH4. **(A)** Adult MDD *vs.* HC. **(B)** Adolescent MDD *vs.* HC. **(C)** Adult MDD *vs.* adolescent MDD. ROC, receiver operating characteristic; MDD, major depressive disorder; HC, healthy control; AUC, area under the curve.

ROC curve analysis indicated that plasma ITIH4 exhibited an AUC value of 0.824 for differentiating adult MDD patients from HCs (specificity = 98.44%, sensitivity = 69.23%; **Figure 1**). Meanwhile, plasma ITIH4 also could provide an acceptable AUC value > 0.700 to identify adolescent MDD patients from HCs (AUC = 0.729; specificity = 98.44%, sensitivity = 54.23%; **Figure 1**). However, for distinguishing adult MDD patients from adolescent MDD patients, plasma ITIH4 just showed an AUC value of 0.646 (specificity = 68.63%, sensitivity = 58.46%; **Figure 1**).

### Influence of antidepressive treatment

After 14-days’ antidepressive treatment, both adult MDD patients and adolescent MDD patients showed significantly reduced HAMD-24 and SDS scores as compared to assessment scores before treatment (**Supplementary Table 1**). Meanwhile, compared with before treatment, plasma GFAP also displayed a significantly decreased levels in both adult MDD and adolescent MDD patients after treatment (**Supplementary Table 2**).

**Figure 2:**
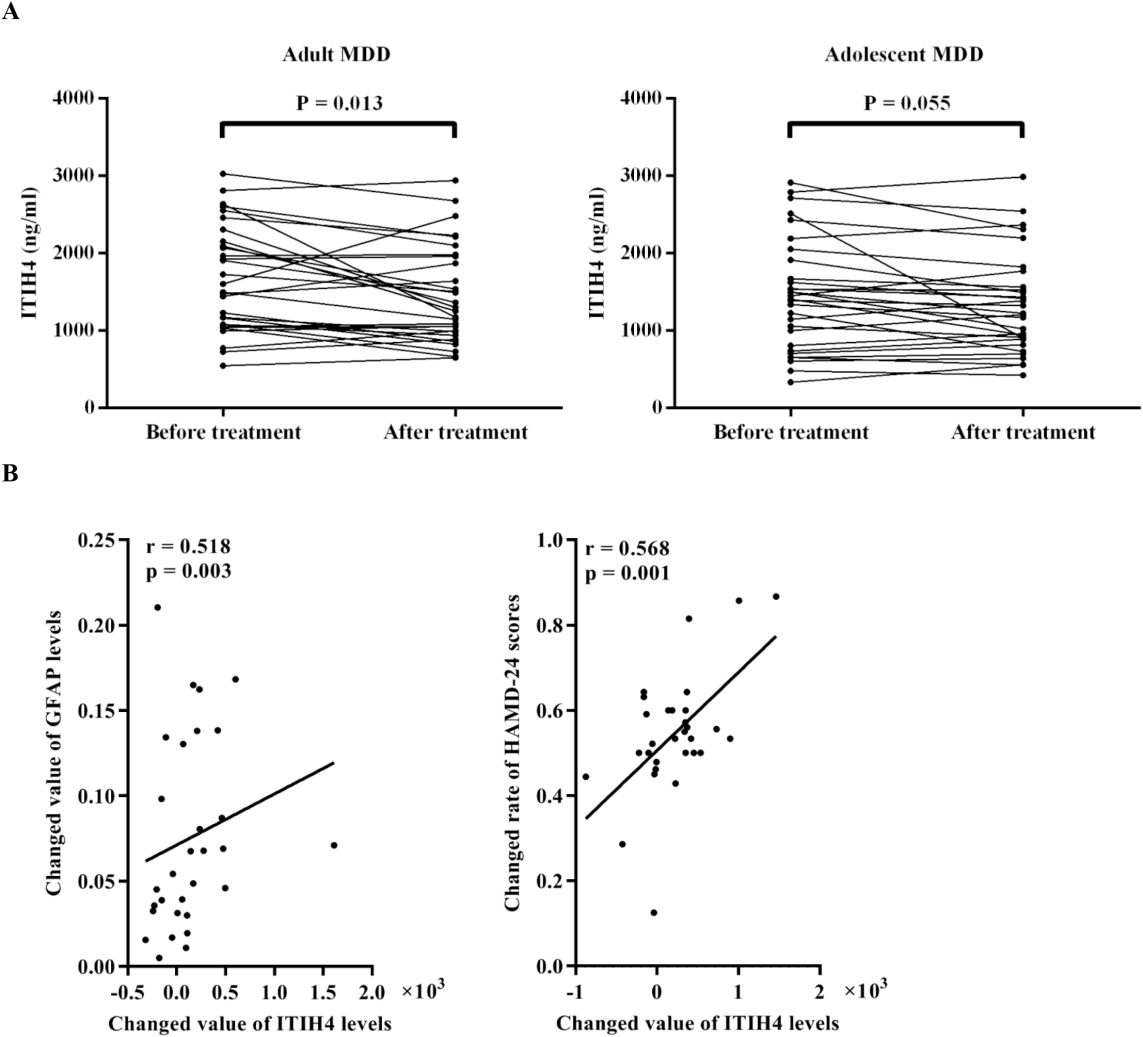
Effect of antidepressive treatment in MDD patients. **(A)** The change of levels of plasma ITIH4 following antidepressive treatment in adult MDD patients and adolescent MDD patients. **(B)** Associations of change of plasma ITIH4 levels with change of plasma GFAP levels and HAMD-24 scores in adult MDD patients. MDD, major depressive disorder; ITIH4, inter-alpha-trypsin inhibitor heavy chain 4; GFAP, glial fibrillary acidic protein; HAMD-24, 24-item Hamilton Depression Scale.

In addition, in the adult MDD group, plasma levels of ITIH4 had a significantly decrease after antidepressive treatment when compared to before treatment (**Figure 2A**). However, there was only a trend in the decrease in the adolescent MDD group before and after treatment (**Figure 2A**).

Further correlation analyses showed that there was a positive correlation between changed value of ITIH4 levels and changed value of GFAP levels in adult MDD patients (annotation: changed value of level = before treatment level – after treatment level) (**Figure 2B**). Meanwhile, in adult MDD patients, the changed rate of HAMD-24 scores was positively correlated with the changed value of plasma ITIH4 levels after treatment (annotation: changed rate of score = [before treatment score – after treatment score]/before treatment score) (**Figure 2B**). However, no any correlation was detected in the adolescent MDD group.

## Discussion

The main findings of the present study are as follows: (1) there was significantly increased levels of ITIH4 in plasma samples of MDD patients compared with HCs, and the significant increase in plasma ITIH4 was observed in adult MDD patients compared with adolescent MDD patients; (2) levels of plasma ITIH4 was positively correlated with HAMD-24 scores and plasma GFAP levels in MDD patients, but the significant correlation between plasma ITIH4 levels and age just was found in adult MDD patients; (3) plasma ITIH4 could provide a good performance to differentiate either adult MDD patients or adolescent MDD patients from HCs; (4) plasma levels of ITIH4 significantly changed following antidepressive treatment in adult MDD patients, but not in adolescent MDD patients; (5) the change in plasma ITIH4 following antidepressive treatment reflected the change in depressive symptoms following treatment in adult MDD patients. Taken together, plasma ITIH4 may be meaningfully associated with depressive symptomatology and therapeutic effect in MDD patients, which showed an obvious age-specific performance.

In consistent with our previous study (17), this study also found significant increase in plasma ITIH4 levels in adult MDD patients as compared to HCs. Meanwhile, the present study firstly found that plasma ITIH4 levels in adolescent MDD patients were significantly higher than HCs but significantly lower than adult MDD patients, which indicated that the plasma levels of ITIH4 showed significant changes in multiple types of MDD when compared to HCs, and these changes displayed an age-specific performance in MDD. Furthermore, our association analyses showed that there was positive correlation between plasma ITIH4 levels and age in adult MDD patients not in adolescent MDD patients, which suggested that age had a key effect on the plasma levels of ITIH4 in adult MDD. However, a smaller age span (*i.e.*, 12-17 years old) used in adolescent MDD patients might be a primary cause to affect the results in the association analysis. Moreover, the interaction between plasma ITIH4 levels and age could be associated with the HAMD-24 scores of adult MDD patients, further suggesting the depressive symptoms could be affected by the plasma ITIH4 levels and age. Although age of onset has been determined as an important element to impact on symptomatology of MDD (25), many pathological differences in MDD may are dependent on the age, *e.g.*, brain gray matter volume and cortical thickness (26) and urinary metabolite expression (27). In the current clinical practice, the same diagnostic criteria of MDD are used in adults and adolescents, which seems to imply the age-independent presentation of the disorder, however, there are explicit aetiological differences between adolescent and adult MDD (28, 29). The differences in plasma ITIH4 expression in the present study between adolescent MDD and adult MDD might indicate a different progress of some molecular changes in the development of adolescent and adult MDD. Additionally, in the present study, our findings provide clinical evidence for the diagnosis of MDD using plasma ITIH4. Plasma ITIH4 provided the great performance for identifying MDD (either adult MDD or adolescent MDD) patients from HCs. However, for distinguishing adult MDD patients from adolescent MDD patients, plasma ITIH4 just provided an inadequate diagnostic power. Therefore, we successfully identified different plasma ITIH4 levels in adolescent and adult MDD patients, which was helpful for developing age-specific diagnostic method for MDD.

The present study found that effective antidepressive drug could change the levels of plasma ITIH4 in adult MDD, which was similar to our previous study (20). However, for adolescent MDD patients, the changes of plasma ITIH4 levels were not significant between before and after treatment. Previous study revealed that differences in treatment influence in adolescent and adult MDDs may be associated with differences in depression symptom profiles, especially vegetative symptoms (30). Our findings further indicated that the potential ITIH4-related pathophysiological mechanisms might be involved in the age-related differences in effect sizes of antidepressant in MDD (31, 32). Additionally, in adult MDD patients, there was a positive correlation between the changed value of plasma ITIH4 levels and the changed rate of HAMD-24 scores following treatment, suggesting the change in plasma ITIH4 could be associated with the therapeutic effect of antidepressive treatment. Hence, plasma ITIH4 might have a potential clinical value for the antidepressive treatment, in particular for adult MDD patients.

Astrocytes play a key role in regulating synaptic and intracellular levels of neurotransmitters and neuroinflammatory processes, and animal and human postmortem studies have demonstrated prominent astrocyte pathology in the pathogenesis of MDD (33, 34). For investigating the possible mechanism of ITIH4 in the pathophysiology of MDD, we detected two robust fluid markers for reflecting astrocyte reactivity, *i.e.*, GFAP and S100β, both of which has generally been found to be increased in MDD (35–38). In the present study, there were positive correlations between plasma GFAP and ITIH4 in both adult and adolescent MDD patients and positive correlations between plasma S100β and ITIH4 in adult MDD patients. Meanwhile, line regression analyses also indicated that potential interaction of plasma GFAP and ITIH4 levels could affect the scores of HAMD-24 in adult MDD patients. These findings suggested that ITIH4-related pathological mechanisms in MDD might be associated with activation of astrocytes (39, 40). Furthermore, through the 14-days’ antidepressive treatment, the change of plasma ITIH4 and GFAP showed a significant correlation in adult MDD patients. Previous studies indicated that the alteration of GFAP expression after antidepressive therapy implied the improvement of the number and morphology of astrocytes in MDD (33, 41, 42). Therefore, the influence of antidepressive treatment on the change of plasma ITIH4 might be associated with the reversibility of change of astrocytes in MDD.

In the present study, plasma ITIH4 was recommended is an acceptable biomarker for the diagnosis of adult or adolescent MDD from HCs. However, this indicator was not splendid biomarker for distinguishing adult MDD patients from adolescent MDD ones. Furthermore, high specificity and low sensitivity of plasma ITIH4 leaded to this indicator just showed moderate diagnostic power for MDD. Our findings provided valuable evidence supported that plasma ITIH4 as a great potential biomarker to combine other markers for establishing the clinically diagnostic model in MDD.

However, the present study has several limitations: (1) the expression of the plasma ITIH4 has not been validated in patients with geriatric depression; (2) the same antidepressant drug is not used during treatment in each MDD patient; (3) animal experiments is lacking to determine the change of astrocytes when ITIH4 expression was up-regulated or down-regulated; (4) due to some patients rejected to provided blood sample secondly at the end of treatment, the change of plasma ITIH4 in MDD before and after treatment needed to further be verified. Thus, further researches, including expanding verification population of MDD patients with multiple ages, strict intervening measures for antidepressive treatment, and depressive-like animal model experiments, are currently ongoing.

## Conclusion

The present study demonstrated that plasma ITIH4 levels were significantly increased in MDD patients as compared to HCs, and adult MDD patients showed significantly higher plasma ITIH4 levels than adolescent MDD patients, suggesting the increase of plasma ITH4 in MDD patients might exhibit an age-related change. Meanwhile, in MDD patients, plasma ITIH4 showed obvious age-specific characteristic in associations of depressive symptomatology, astrocyte-related molecular changes, and effect of antidepressive treatment. The present findings may enhance clinical evaluations for MDD and assist clinicians in promoting earlier detection and individualized treatment in MDD individuals.

## Data Availability

The original contributions presented in the study are included in the article/**Supplementary Material**. Further inquiries can be directed to the corresponding authors.
