# Supplementary material for: Plasma alpha-trypsin inhibitor heavy chain 4 as an age-specific biomarker in the diagnosis and treatment of major depressive disorder
Source: Front Psychiatry. 2024 Sep 11;15:1449202. doi: 10.3389/fpsyt.2024.1449202 (PMC11422199; doi:10.3389/fpsyt.2024.1449202)
Supplement: Supplementary file 1 [file Table1.docx]

**Sample size calculation**

The sample size calculation was performed using an online sample size calculators (<https://sample-size.net/>). We performed the sample size calculation (α=0.05, β=0.2) to confirm whether the cohorts (65 adult MDD patients, 51 adolescent MDD patients, and 64 healthy controls) was acceptable based on the measurement results of plasma ITIH4. The results of sample size calculation are as following:

adult MDD *vs.* adolescent MDD: N_total_ = 96, N_group 1_ = 48, N_group 2_ = 48, Power = 0.805;

adult MDD *vs.* HCs: N_total_ = 16, N_group 1_ = 8, N_group 2_ = 8, Power = 0.847;

adolescent MDD *vs.* HCs: N_total_ = 42, N_group 1_ = 21, N_group 2_ = 21, Power = 0.812;

Therefore, the present sample size (65 adult MDD patients, 51 adolescent MDD patients, and 64 healthy controls) can provide an acceptable power (> 80%), and the sample size using in the present study can meet the minimum sample requirement based on results of the sample size calculation.

**Supplementary Table 1. The change of psychological assessments in adult MDD participants and adolescent MDD participants after antidepressive treatment.**

|  | Adult MDD (N = 65) | | Adolescent MDD (N = 51) | |
| --- | --- | --- | --- | --- |
|  | After treatment | P-value^*^ | After treatment | P-value^*^ |
| HAMD-24 score | 8.77 ± 3.08 | < 0.001 | 9.47 ± 3.68 | < 0.001 |
| SDS score | 53.18 ± 12.99 | < 0.001 | 59.04 ± 13.51 | < 0.001 |

MDD: major depressive disorder; HAMD-24: 24-item Hamilton Depression Scale; SDS: Self-Rating Depression Scale.

^*^ Paired t-test.

**Supplementary Table 2. The change of plasma indices’ levels in adult MDD participants and adolescent MDD participants after antidepressive treatment.**

|  | Adult MDD (N = 30) | | Adolescent MDD (N = 30) | |
| --- | --- | --- | --- | --- |
|  | After treatment | P-value^*^ | After treatment | P-value^*^ |
| GFAP | 0.15 ± 0.05 | 0.001 | 0.17 ± 0.07 | 0.045 |
| S100β | 100.34 ± 19.86 | 0.316 | 88.14 ± 18.25 | 0.143 |

MDD: major depressive disorder; GFAP: glial fibrillary acidic protein; S100β, S100beta protein.

^*^ Paired t-test.
